# The Rosetta Phenotype Harmonization Method Facilitates Finding a Relationship Quantitative Trait Locus for a Complex Cognitive Trait

**DOI:** 10.3390/genes14091748

**Published:** 2023-08-31

**Authors:** Stephen A. Petrill, Brett G. Klamer, Steven Buyske, Erik G. Willcutt, Jeffrey R. Gruen, David J. Francis, Judy F. Flax, Linda M. Brzustowicz, Christopher W. Bartlett

**Affiliations:** 1 Department of Psychology, College of Arts and Sciences, The Ohio State University, Columbus, OH 43210, USA; petrill.2@osu.edu; 2 Center for Biostatistics, The Ohio State University, Columbus, OH 43210, USA; brett.klamer@osumc.edu; 3 Department of Statistics, Rutgers, The State University of New Jersey, Piscataway, NJ 08854, USA; buyske@stat.rutgers.edu; 4 Department of Psychology, University of Colorado Boulder, Boulder, CO 80309, USA; erik.willcutt@colorado.edu; 5 Departments of Pediatrics and of Genetics, Yale Medical School, New Haven, CT 06511, USA; jeffrey.gruen@yale.edu; 6 Texas Institute for Measurement, Evaluation, and Statistics, University of Houston, Houston, TX 77004, USA; dfrancis@central.uh.edu; 7 Department of Genetics, Rutgers, The State University of New Jersey, Piscataway, NJ 08854, USA; judyflax17@gmail.com (J.F.F.); lbrz@hginj.rutgers.edu (L.M.B.); 8 The Steve & Cindy Rasmussen Institute for Genomic Medicine in the Abigail Wexner Research Institute at Nationwide Children’s Hospital, Columbus, OH 43205, USA; 9 Department of Pediatrics, College of Medicine, The Ohio State University, Columbus, OH 43205, USA

**Keywords:** data harmonization genetics, latent traits, missing data, brain-derived neurotrophic factor, working memory, reading comprehension

## Abstract

Genetics researchers increasingly combine data across many sources to increase power and to conduct analyses that cross multiple individual studies. However, there is often a lack of alignment on outcome measures when the same constructs are examined across studies. This inhibits comparison across individual studies and may impact the findings from meta-analysis. Using a well-characterized genotypic (brain-derived neurotrophic factor: BDNF) and phenotypic constructs (working memory and reading comprehension), we employ an approach called Rosetta, which allows for the simultaneous examination of primary studies that employ related but incompletely overlapping data. We examined four studies of BDNF, working memory, and reading comprehension with a combined sample size of 1711 participants. Although the correlation between working memory and reading comprehension over all participants was high, as expected (ρ = 0.45), the correlation between working memory and reading comprehension was attenuated in the BDNF Met/Met genotype group (ρ = 0.18, n.s.) but not in the Val/Val (ρ = 0.44) or Val/Met (ρ = 0.41) groups. These findings indicate that Met/Met carriers may be a unique and robustly defined subgroup in terms of memory and reading comprehension. This study demonstrates the utility of the Rosetta method when examining complex phenotypes across multiple studies, including psychiatric genetic studies, as shown here, and also for the mega-analysis of cohorts generally.

## 1. Introduction

Comparisons of primary data across studies, as well as meta-analysis, have difficulty effectively managing the variability measurement that naturally occurs across primary studies [1,2,3]. This variability can be substantial, involving nonoverlapping constructs across studies but also differences in measurement of the same constructs. Often, these differences can be subtle [4] but nevertheless meaningful, especially when making conclusions from a large literature for their theoretical or clinical applications. The resulting lack of alignment of measurement across studies has the potential to distort meta-analytic studies and can also force difficult decisions for researchers trying to conduct joint primary analyses across multiple datasets [4].

For example, brain-derived neurotrophic factor (BDNF) has drawn particular attention in the literature for its wide-ranging effects on the central nervous system, including significant associations with schizophrenia [5,6,7], depression [8] and anxiety [9,10], mood disorders [11,12], bipolar disorder [13,14], and cognition [15,16]. Subsequent work has begun to elucidate the neurobiological and proteomic pathways of BDNF [17,18,19,20,21]. Most of these studies focus on SNP rs6256, a functional single nucleotide polymorphism which results in a valine to methionine substitution (val66met), leading to reduced mature BDNF expression [22]. These statistical relationships often demonstrate moderator effects on phenotypes or statistical interactions and perhaps even epistatic interactions as opposed to main effects [8,23,24,25]. There are numerous meta-analyses of BDNF [5,8,16,26,27], which is the preferred method of comparing results across studies.

Though highly useful and informative, meta-analysis has some limitations. First, meta-analysis does not directly assess person-level variability, instead examining variability across the selected studies. More fundamentally, meta-analysis, as well as most comparisons of primary data across studies, has difficulty effectively managing the variability measurement that naturally occurs across primary studies [1,2,3]. This variability can be substantial, involving nonoverlapping constructs across studies but also differences in measurement of the same constructs. Often, these differences can be subtle [4] but nevertheless meaningful, especially when making conclusions from a large literature for their theoretical or clinical applications. The resulting lack of alignment of measurement across studies has the potential to distort meta-analytic studies and can also force difficult decisions for researchers trying to conduct joint primary analyses across multiple datasets [4].

In the case of BDNF, meta-analysis limitations impinge on clinical studies, especially given that the relationships among BDNF and complex human traits appear to be moderated effects as opposed to main effects. This is true for all meta-analyses that compare the impact of gene variants across multiple studies. This is especially true when allelic or genotypic frequencies are uncommon or rare. Single studies often have reduced power to study uncommon and rare variants. When a variant is observed by independent research groups, a method that best combines data across those groups is desirable.

The present study addresses two goals. First, we employ a novel analytical approach that uses a flexible platform to examine the relationships among gene variants and phenotypes across multiple studies using primary data. This method is called Rosetta [4] and it is freely available as the *rosettaR* package v 0.1 for the R programming language (https://github.com/cwbartlett/rosettaR (accessed on 23 June 2023)). We apply this method to four datasets that had genotype data and our cognitive phenotypes of interest to assess the effects of BDNF on complex cognition. Second, we compare the results of meta-analysis to Rosetta and highlight the difference between the two approaches for combining data. Overall, the current analysis examines five independent studies to further define the relationships among BDNF, memory, and reading comprehension. This study demonstrates the usefulness of the Rosetta approach, which not only provides a powerful tool for examining genetic effects across different studies and multiple measurement domains but is also applicable to nongenetic studies. This study also tests a hypothesis that is important to the language genetics literature. Namely, we hypothesize that language and reading measures are correlated within the Val/Val and the Val/Met BDNF groups but not within the Met/Met group.

## 2. Materials and Methods

To combine data, we used a latent trait approach, which refers to a method that analyzes an unobserved (latent) trait based on observable or measured behavioral responses. This approach was used to create a set of analyzable traits that represent each underlying phenotypic construct, where each latent trait is on the same scale within the construct and across datasets. This approach, called Rosetta, allows for a joint analysis across all datasets, often called a mega-analysis. While our focus is on psychiatric genetics and the example dataset focuses within this domain (i.e., memory and reading comprehension), we only used those data as a real-world example of how Rosetta can be applied to genetic datasets with varied phenotypes across datasets. The use of latent factor modeling has the advantage of (1) generating a common metric for the analysis of each construct that we then used for a single mega-analysis and (2) reducing measurement error through the combination of multiple measures of each construct. Each study was also analyzed separately to assess patterns across the datasets.

Overview of the Rosetta method [4]: Rosetta is an analytical framework that utilizes the underlying mathematics of confirmatory factor analysis (CFA) to process data. Differing from traditional CFA, Rosetta does not perform hypothesis testing, instead employing the same math to apply constraints for the linear combination of weights applied to phenotypes, ultimately deriving a set of latent factors. The algorithm’s novelty lies in its ability to handle partially overlapping datasets. Rosetta estimates correlations between all measures, performing eigendecomposition for factor loadings and communalities. Structural equation modeling (SEM) is then applied to maintain correlation constraints across datasets, ensuring consistent factor equality across datasets. The resultant factor scores represent the same underlying latent factor, where the datasets can be combined for comprehensive analysis.

As an overview of the study methods, we combined data from four independent studies including measures for the behavioral constructs of working memory and reading comprehension along with genetic data for *BDNF*. To combine data, we used Rosetta to create a set of latent traits for each underlying construct (i.e., memory and reading comprehension). Each latent trait is on the same scale across datasets. Within each study, working memory and reading comprehension were assessed with (partially) nonoverlapping measures (Table 1). The use of latent factor modeling has the advantage of both generating a common metric for the analysis of each construct that we used for a single mega-analysis and also reducing measurement error through the combination of multiple measures of each construct. Each study was also analyzed separately to assess patterns across the datasets. Table 1 shows the specific measures, by study, for memory and reading comprehension included in the respective latent factors. We tested the correlation of reading comprehension and memory by *BDNF* genotypes (i.e., three genotypic groups). A permutation test was implemented to assess if the observed pattern of correlations was significant.

### 2.1. Component Studies

We combined data from four independent studies including measures for the behavioral constructs of working memory and reading comprehension along with genetic data for *BDNF*. Within each study, working memory and reading comprehension were assessed with (partially) nonoverlapping measures (Table 1). Table 1 shows the specific measures, by study, for memory and reading comprehension included in the respective latent factors.

*The Western Reserve Reading and Math Project (WRRMP)* is an ongoing longitudinal twin study consisting of a community-based sample of both monozygotic and same/opposite sex dizygotic twins followed for over 10 years beginning at age 5 [28,29].

*The New Jersey Language-Autism Genetic Study (NJLAGS)* is a collection of nuclear and extended families that segregate both autism and specific language impairment in each pedigree [30,31].

*The Biology of Language Study (BLS)* is an ongoing study of the genetics of specific language impairment conducted in 31 nuclear and extended pedigrees ascertained for multiple family members with language impairment [32,33].

*The Colorado Learning Disabilities Research Center (CLDRC)* is an ongoing collection of monozygotic and dizygotic twins ascertained for reading disability, attention-deficit/hyperactivity disorder, and other learning disabilities [34]. Study name, sample sizes, and SNP-genotyping platform for each component study can be found in the online supplement (Table S1).

### 2.2. Statistical Analysis

We conducted all statistical analysis with the R statistical language v4.2.1 [35] in the RStudio integrated development environment v2022.07.2 [36] with the standard libraries unless otherwise noted. We assessed Hardy–Weinberg equilibrium for BDNF genotypes per study using the standard χ^2^ test implemented in *HardyWeinberg* package v1.7.2 [37]. Assessing the equality of genotype frequencies across all studies was also conducted with a χ^2^ test. To test the main effects of *BDNF* SNP rs6265, we conducted linear regression analysis assuming an additive allelic effect. Since persons in the same family are correlated observations, to account for familial relationships, we implemented a repeated sampling across family units to maintain independence of observations while also adjusting for error in estimates.

To combine datasets, we used the Rosetta method [4] to create two common latent traits, one for working memory and one for reading comprehension. Rosetta creates latent traits using the modified factor analytic method described elsewhere [4]. Briefly, Rosetta processes datasets that have imperfectly matched measurements of the same underlying constructs. In the usual application of Rosetta, the required correlation matrix input for the factor analysis is calculated using all possible slices across the datasets to create a complete pairwise correlation matrix while maximizing the sample size per slice. In the present case, NJLAGS had a unique reading comprehension measure (i.e., a measure that appears only in one study, see Table 1). In this case, an additional numerical procedure was included to complete the pairwise correlation matrix. The matrix was numerically evaluated to assess if it was positive definite (PD, a requirement for the linear algebra of factor analysis whereby matrices must have eigenvalues > 0) at each iteration. The Frobenious distance between the current matrix and the nearest PD matrix using Higham’s method was minimized [38], requiring convergence to be achieved in less than 500 iterations. The function was optimized using the limited-memory Broyden–Fletcher–Goldfarb–Shanno optimization algorithm that is commonly applied in numerical statistics and implemented in the *optim* function from the standard R *stats* package.

Rosetta allows the input of a preferred factor structure, if such information is known from the literature. In Table 1, we show our chosen factor constituents by dataset. All previously identified, highly related measures available in each dataset were included in each factor. To help scale the reading comprehension factor for NJLAGS, we added an additional related factor with overlap between datasets, including single-word reading and math problem-solving.

Within each dataset, we analyzed correlations between the Rosetta output factors for memory and reading comprehension stratified by genotype using the standard Pearson’s product-moment correlation by genotype.

To assess the null hypothesis that all three *BDNF*-genotype subsets have the same correlation as when all three genotypes are analyzed jointly, we performed a parametric bootstrap analysis by dataset. Using the observed working memory and reading comprehension data, we randomly selected subjects without replacement to subgroups of size equal to the original *BDNF*-genotype subsets. Correlations were calculated by subgroup for each bootstrap replicate (in the same way as the original real analysis). The value of the correlation by subgroup was collated into a file for further analysis in the next step, for a total of 10,000 bootstraps (10,000,000 to assess all studies combined). Next, we calculated the standard deviation of the three correlations per replicate to assess the distribution of deviations assuming no effect of the genotype. The permutation subgroups were chosen randomly without regard to any genetic information, which necessarily assumes the correlation is constant between the subgroups. This empirical distribution can be used as the basis for null hypothesis testing by comparing the observed difference in correlations across genotypes with the distribution derived from the bootstraps. In this case, the *p*-value represents how often a deviation equal to, or larger than, the observed deviation is expected by chance if BDNF had no effect on the correlation between memory and reading. We used *p* < 0.05 as the cut-off for significance.

## 3. Results

To examine the relationship between BDNF and assessments of working memory and reading comprehension across the WRRMP, NJLAGS, BLS, and CLDRC studies, we analyzed the genotype at SNP rs6256. To test if BDNF had a main effect on working memory and/or reading comprehension, the Rosetta output for working memory and reading comprehension factors were regressed onto *BDNF* rs6265 genotypes (Table 2). Genotype quality control was conducted within each study (see Methods) and each of the four studies—WRRMP, NJLAGS, BLS, and CLDRC—were in Hardy–Weinberg equilibrium (*p* > 0.05). No regressions of either working memory or reading comprehension on rs6265 were significant either by dataset or in the mega-analysis of all datasets.

Next, to test whether our initial observation of a weak or absent correlation between reading comprehension and working memory for BDNF Met/Met carriers in Canadian families of Celtic ancestry could be replicated, we examined correlations between the four studies. In all datasets, the individuals with the rs6265 genotype leading to homozygous Met/Met lack a significant correlation between the reading comprehension and working memory measures, while those measures are significantly correlated in individuals with the other genotypes (just as in the analysis of all genotypes jointly). The results also hold for all samples in the analysis (see All Samples in Table 3). Given the pattern of correlation between working memory and reading comprehension for individuals with Val/Val and Val/Met variants but not for individuals with Met/Met variants, we next sought to assess how rarely this configuration occurs under the null hypothesis.

Assuming no effect of rs6265 on the correlation of reading comprehension and working memory, a permutation procedure can be used to derive an empirical null distribution. Random subsets of subjects are expected to have the same correlation on average as the dataset from which they were randomly selected. This assertion can be used to derive the counterfactual for this study. If we assume that the rs6265 T/T genotype (corresponding to Met/Met) has the same correlation as the other two genotypically defined groups, we can calculate the probability that only one genotype out of the three would randomly have a reduced correlation through parametric bootstrapping (results are shown in Table 4). The permutation *p*-values by study all indicate that the pattern of correlations is rare (i.e., C/C and C/T groups are correlated but T/T is not) and even with Bonferroni correction (*p* < 0.01), all four studies are significant. When assessing all samples combined, the permutation test was likewise significant (*p* < 4 × 10^−4^).

## 4. Discussion

This study was designed to demonstrate how Rosetta can be applied to a common problem in consortium data analysis, namely, the situation where a researcher seeks a larger sample size by combing datasets that do not have identical measurement strategies. This situation is not unique to genetics, though this study includes a demonstration from that domain. One goal of this work was to continue to adapt the *rosettaR* package to real-world situations, such as the complication mentioned in the Methods section detailing how the differing measurement strategies across studies can induce numerical complications, which the *rosettaR* package can now handle. Working on real data also highlights a practical matter, in that Rosetta scores are on a common scale so graphing all component studies on one graph greatly highlights the effect being studied.

In terms of the types of data where Rosetta is likely to be helpful, its foundation is that of latent trait analysis, so the same guidelines are practical to employ in this context [39]. (1) A sample size of at least 300 is often suggested, and in the context of genetics, this is not an issue since larger samples are needed for almost all study designs. (2) The correlation of phenotypes within each latent trait and also the correlation between latent traits should be significant and replicable. Ideally, the average within correlations is greater than 0.3 and between correlations may be lower. There are not strict cut-offs as each cluster of phenotypes in a real setting will have other subtitles to consider, but it is clear that the phenotypes across studies are not highly correlated; then, any analysis, including both meta-analysis and Rosetta, would not be reasonable. (3) The correlation across the phenotypes needs to be estimable from the data or leverage outside data such as published norms. Unique situations where no measurement overlaps across any study would not allow correlations to be calculated and would not allow Rosetta to be applied. Given these general conditions, Rosetta may offer modeling advantages over meta-analysis.

With respect to *BDNF*, genotypes underlying Val/Val and Val/Met groups show correlations between working memory and reading comprehension, but we found no evidence for correlation in individuals with Met/Met variants in any single dataset, nor in the combined dataset. Variants that mediate the correlation between gene transcription traits have been observed previously [40] (often called relationship quantitative trait loci or rQTLs [41]), but finding this genetic mechanism for modulating the relationship between two human cognitive quantitative traits is novel. Further research is required to disentangle BDNF-associated working memory as either (1) a support for reading comprehension in general versus (2) a possible compensation mechanism for reading comprehension-related deficits that is not available for persons that are Met/Met but is available for persons that are Val/Val or Val/Met. This is a recessive pattern of inheritance, whereby the effect is only seen with two copies of Met and no effect is seen with only one copy of Met.

Taken together, the conservative interpretation of our findings is that BDNF Met/Met attenuates the correlation between reading comprehension and memory such that a correlation was not detectable in our study, if indeed the correlation is greater than zero. Larger studies using Rosetta to combine datasets, and possibly meta-analyses when the primary data are not available, will be required to estimate BDNF Met/Met attenuation with greater precision, or to conclude that the correlation is truly negligible (or zero). Our research suggests that Met/Met carriers are a unique and robustly defined subgroup in terms of memory and comprehending written language. Further studies of this subgroup will elucidate the role of BNDF-associated memory in oral and written language learning. Though more research will be needed to explain how BDNF-associated memory resolves between reading comprehension and working memory, this study provides further evidence for the existence of BDNF-dependent memory processes in human cognition, a topic that may rapidly develop since animal models are already available.

## 5. Conclusions

This study demonstrates the versatile capabilities of Rosetta in assessing relationships between gene variants and cognitive phenotypes across multiple studies, offering an innovative approach to consolidating disparate datasets. Despite the complexities presented by varying measurement strategies, Rosetta proves adept in handling these numerical complications, allowing for the integration of all study components into one graph for enhanced interpretation and one model for modeling flexibility. Given the appropriate sample size and correlation conditions, Rosetta could serve as an alternative model to traditional meta-analysis, enhancing the scope of genetic studies and facilitating the exploration of nuanced trait correlations [16].

## Figures and Tables

**Table 1:** Measures grouped by latent factor and dataset.

Factor	Measure	WRRP	NJLAGS	BLS	CLRDC
Working Memory Factor	CLEF Recalling Sentences	X *	X		
	CTOPP Nonword Repetition	X	X	X	
	Memory for Digits	X			
	Digit Span	X		X	X
	Coris Block	X			
	CTOPP Ellision	X	X	X	
	Memory for Sentences	X		X	
	Backward Span (35)				
	Backward Span (58)				
	Backward Span (90)				
	Backward Span (Sum)				
Reading Comprehension Factor	Passage Comprehension (Woodcock)	X		X	
	Reading Comprehension PIAT	X			X
	Gray Oral Reading Test (Comprehension) **		*X*		
Math/Reading Factor	WJ Word Attack	X	X	X	
	WJ Word Identification	X	X	X	
	*WJ Applied Problems*	*X*			

* X indicates a measure is in the given dataset. ** Measures unique to a study are italicized.

**Table 2:** Regression of BDNF SNP rs6265 on cognitive traits.

Dataset	Trait	*R* ^2^	*p*-Value
WRRP	Reading Comprehension	0.0	0.30
	Memory	0.0	0.45
NJLAGS	Reading Comprehension	0.0	0.12
	Memory	0.0	0.46
BLS	Reading Comprehension	0.0	0.79
	Memory	0.0	0.59
CLRDC	Reading Comprehension	0.0	0.28
	Memory	0.0	0.93
All Samples	Reading Comprehension	0.0	0.92
	Memory	0.0	0.06

**Table 3:** Correlations between latent traits for working memory and reading comprehension by subset and genotype.

Sample	Statistic	All Genotypes	Val/Val C/C	Val/Met C/T	Met/Met T/T
WRRP	Correlation	0.36	0.41	0.37	−0.14
	N	290	177	96	17
	*p*-value	*4 × 10^−10^*	*9 × 10^−9^*	*2 × 10^−4^*	0.57
NJLAGS	Correlation	0.58	0.63	0.56	0.23
	N	334	203	110	21
	*p*-value	*1 × 10^−33^*	*3 × 10^−24^*	*9 × 10^−11^*	0.31
BLS	Correlation	0.45	0.42	0.53	0.12
	N	*320*	219	92	9
	*p*-value	*1 × 10^−17^*	*4 × 10^−11^*	*3 × 10^−8^*	0.76
CLRDC	Correlation	0.26	0.27	0.26	−0.09
	N	*767*	*506*	*228*	33
	*p*-value	*1 × 10^−13^*	*1 × 10^−10^*	*6 × 10^−6^*	0.61
All Samples	Correlation	0.45	0.44	0.41	0.18
	N	1711	886	434	71
	*p*-value	*3 × 10^−85^*	*1 × 10^−41^*	*1 × 10^−18^*	0.13

Significant *p*-values are italicized.

**Table 4:** Permutation tests by subset and combined.

Dataset	N	Permutation *p*-Value	Avg “val/val”	Avg “val/met”	Avg “met/met”
WRRP	290	0.0023	0.356	0.355	0.349
NJLAGS	334	0.0006	0.582	0.579	0.559
BLS	320	0.0112	0.453	0.448	0.415
CLRDC	767	<0.0001	0.263	0.268	0.321
ALL	1711	4 × 10^−4^	0.42	0.42	0.43

## Data Availability

Component datasets are available from the individual study PIs, relevant NIH archives, or both. Data used specifically in this study are available upon request.

## Supplementary Materials

The following supporting information can be downloaded at: https://www.mdpi.com/article/10.3390/genes14091748/s1, Table S1: component dataset characteristics.
