# Supplementary material for: The Rosetta Phenotype Harmonization Method Facilitates Finding a Relationship Quantitative Trait Locus for a Complex Cognitive Trait
Source: Genes (Basel). 2023 Aug 31;14(9):1748. doi: 10.3390/genes14091748 (PMC10531321; doi:10.3390/genes14091748)
Supplement: Supplementary file 1 [file genes-14-01748-s001.zip › genes-2543771-supplementary.pdf]

Supplemental Table 1. Component dataset characteristics

Dataset	N	Subjects	Genotyping
WRRMP	290	unrelated	PsychArray v1.1
NJLAGS	334	nuclear families	Affymetrix Axiom v1.0 microarray PsychArray v1.1
BLS	320	nuclear and extended pedigrees	PsychArray v1.1
CLDRC	767	unrelated	Illumina Human OmniExpress genotyping panel
